# Applicability of RECIST1.1, MDACC and SPINO Tumor Response Criteria After Adjuvant Stereotactic Spinal Radiosurgery in Surgically Treated Spinal Metastases

**DOI:** 10.3390/medicina62071253

**Published:** 2026-06-29

**Authors:** Francisco Alfredo Call-Orellana, Juan Pablo Zuluaga-Garcia, Romulo Augusto Andrade de Almeida, Alex Beck, Thomas H. Beckham, Amol Ghia, Jing Li, Martin C. Tom, Mary Frances McAleer, Subha Perni, Chenyang Wang, Debra N. Yeboa, Kevin A. Cross, Christopher A. Alvarez-Breckenridge, Gil Kimchi, Laurence D. Rhines, Claudio E. Tatsui, Robert Y. North

**Affiliations:** 1 Department of Neurosurgery, The University of Texas MD Anderson Cancer Center, Houston, TX 77030, USA; facall@mdanderson.org (F.A.C.-O.); jpzuluaga@mdanderson.org (J.P.Z.-G.); kacross@mdanderson.org (K.A.C.); calvarez11@mdanderson.org (C.A.A.-B.); gkimchi@mdanderson.org (G.K.); lrhines@mdanderson.org (L.D.R.); cetatsui@mdanderson.org (C.E.T.); 2 Department of Neurosurgery, Lahey Hospital, Burlington, MA 01805, USA; romulo.andradedealmeida@lahey.org; 3 Baylor College of Medicine, Houston, TX 77030, USA; alex.beck@bcm.edu; 4 Department of Radiation Oncology, The University of Texas MD Anderson Cancer Center, Houston, TX 77030, USA; thbeckham@mdanderson.org (T.H.B.); ajghia@mdanderson.org (A.G.); jing.li@mdanderson.org (J.L.); mtom@mdanderson.org (M.C.T.); mfmcalee@mdanderson.org (M.F.M.); sperni@mdanderson.org (S.P.); cwang23@mdanderson.org (C.W.); dnyeboa@mdanderson.org (D.N.Y.)

**Keywords:** metastasis, spine oncology, stereotactic radiotherapy, tumor response

## Abstract

*Background and Objectives*: Up to 40% of patients with cancer develop spinal metastases, and stereotactic spinal radiosurgery (SSRS) achieves high local control rates as definitive or postoperative treatment. Multiple tumor response assessments have been used but their compared clinical performance in post-surgical patients remains unclear. We sought to compare the applicability of RECIST1.1, MDACC and SPINO criteria. *Materials and Methods*: This IRB-approved retrospective study included patients with high-grade epidural spinal cord compression treated with decompressive surgery followed by adjuvant SSRS, with MRI follow-up available. Lesions were classified according to each of the scale’s objective (RECIST1.1 and MDACC) and subjective (SPINO [radiology reports]) criteria. *Results*: Ninety-four treated levels in 93 patients (median age 58.9 years) were analyzed. Most metastases were thoracic, and all cases had preoperative high-grade epidural spinal cord compression. Adjuvant SSRS was delivered in one or three fractions. Median follow-up was 16 months (range, 1–132), SPINO-based assessment was feasible in 100% of cases, RECIST1.1 in 43.6% and MDACC in 46.8%. Progressive disease criteria were met in 21.3% of cases using SPINO-based assessment, 19.5% using RECIST1.1, and 6.8% using MDACC. *Conclusions*: The SPINO recommendations provide a practical and comprehensive framework for radiographic response assessment in monitoring spinal metastases treated with a combination of surgical decompression and adjuvant SSRS.

## 1. Introduction

Spinal metastatic disease is estimated to affect up to 40% of patients with cancer. Approximately 10% of patients will present with symptoms secondary to epidural spinal cord compression (ESCC) or vertebral body (VB) fracture, such as pain and neurologic deficits, often prompting urgent treatment [1,2].

Management of spinal metastatic disease often requires a multidisciplinary collaboration between specialists in medical oncology, radiation oncology, and spine surgery [3]. Systemically administered anti-neoplastic therapies have an important role in overall control of metastatic disease burden, including spinal metastases, and are an important consideration in management of spinal metastatic disease. Radiotherapy has long demonstrated benefit for local control of spinal metastasis and is useful in treatment of both gross and microscopic diseases. Spine surgery is often performed to address mechanical instability and neurological deficits associated with tumoral compression of the neural elements. Surgical interventions can also be used to affect local control via either complete resection or via “separation surgery” in which spatial margin between the tumor and the spinal cord is created allowing for delivery of ablative radiotherapy via stereotactic spinal radiosurgery (SSRS) [4,5,6,7]. The most common radiotherapy approaches for spinal metastatic disease include fractionated external beam radiotherapy (EBRT) and stereotactic spinal radiosurgery (SSRS) [8]. SSRS is associated with both improved local control and pain outcomes relative to EBRT, achieving local control rates > 90% at 1 year irrespective of tumor histology [9,10,11,12].

Shared terminology is critical in the communication that allows for this multidisciplinary collaboration in management of spinal metastatic disease. A variety of scoring systems and terminologies including the spinal instability neoplastic score (SINS); epidural spinal cord compression scale (ESCC); metastatic spinal tumor frailty index (MSTFI); neurology, oncology, mechanics, and systematics (NOMS) framework; Karnofsky performance status (KPS); Eastern cooperative oncology group (ECOG) scale; ASIA impairment scale, Frankel score, among others, have been created or adopted to create an objective clinical picture of the patient, help guide treatments and understand clinical outcomes [13,14,15,16,17,18,19].

In contrast, there is less robust literature dedicated to post-treatment monitoring/response in spinal metastatic disease despite the critical importance of surveillance during treatment for systemic diseases such as metastatic cancer. Historically, International Union Against Cancer (UICC, 1977) and the World Health Organization (WHO, 1979) published tools that attempted to evaluate this, but were outdated by the widespread use of CT. The Response Evaluation Criteria in Solid Tumors (RECIST) replaced these sets of criteria, and on its 1.1 version they included bone metastasis with soft tissue masses ≥10 mm into consideration as target lesions [20]. MD Anderson Cancer Center (MDACC) published their bone-specific response assessment tool based upon the WHO classification while incorporating CT and MRI into their proposed criteria [21]. RECIST and MDACC classify response into four categories: progressive disease, partial response, complete response, and stable disease [22].

Objective assessment of tumor response to therapy systems provides frameworks for classifying response into defined categories, enabling shared terminology across specialties. consistent comparison across patients, institutions, and clinical trials [23,24]. Though the benefits of such systems are most apparent for research and data sharing, the same systems may also provide direct clinical utility at the individual patient level. Accurate and consistent determinations of treatment response impact clinical decisions for treatment changes including revision surgery, re-irradiation, escalation in systemic therapy, or changes in surveillance intervals. This is especially true in scenarios of difficult-to-interpret imaging anatomy such as postoperative surgical beds, tumors with multicompartmental extension, and imaging distortion due to medical implants. However, despite their widespread use and perceived utility, these systems were not created specifically for spinal metastasis, and did not consider some of the unique aspects of monitoring in spinal metastatic disease such as the involvement of multiple anatomic compartments (bone, paraspinal tissues of neck, trunk, pelvis, epidural and direct neural involvement), post-surgical anatomic distortions, and imaging artifact related to spinal implants [25,26].

In an attempt to create a consensus for evaluation of tumor response after SSRS, the SPINO group (2015) published a set of recommendations assessing tumor response to treatment and considering bone, epidural, and soft tissue metastasis [27]. The SPINO recommendations provide a strong conceptual framework, but they largely rely on qualitative assessment and provide only a binary categorization of local control versus local progression.

Given the coexistence of three scales to assess tumor response and the absence of a widely adopted standard for reporting we evaluated the applicability of RECIST1.1, MDACC criteria, and SPINO group recommendations in a cohort of patients treated with decompressive surgery and adjuvant SSRS for spinal metastatic disease from our institution.

## 2. Materials and Methods

### 2.1. Study Design, Population, and Ethics

This was an IRB-approved (protocol 2021-0766) retrospective study. Electronic medical records of patients treated for spinal metastases by spine surgeons at our institution between 2006 and 2023 were reviewed. Patients were eligible if they had high-grade ESCC (grades 1c, 2, or 3) treated with decompressive surgery and posterolateral segmental stabilization followed by adjuvant SSRS within 3 months, with available follow-up data and MRI, including radiology reports. For those patients with contiguous multivertebral disease, analysis included all levels. Cases of surgically treated discontinuous segments were treated independently.

### 2.2. Imaging Data Analysis, Response Criteria Systems and Classification of Lesions

MRI was the primary imaging modality for response assessment and, together with radiology reports, was used to classify tumor response. Reviewed MRIs included the one taken after surgery and before SSRS, to serve as the baseline, and all subsequent MRIs; generally, every three months during the first year, and with fixed intervals of up to 12 months, depending on response to oncologic treatment and history of stability. All images were independently reviewed except for 3 cases in which MRI images were not available and radiologist report alone was utilized. Imaging reviews were performed by multiple authors (FCO, RAA, AB) and disagreement with radiology reports or across reviewers was reviewed to reach a consensus with final adjudication by senior author (RYN).

Pseudoprogression was defined as equivocal radiology report for progression or MRI findings with transient growth that subsequently ceased on follow-up MRI without further interventions or treatments. If new treatment was initiated, including changes in systemic therapy, re-irradiation, or surgery, this was categorized as progression. MRIs or PET scans were reviewed and any lesions maintaining growth or with high metabolic activity were considered to be local progression. There were no cases of suspected pseudoprogression that could not be appropriately categorized based on subsequent follow-up imaging.

For SPINO-based response assessment, the treated lesion was recorded and categorized as local control or local progression as defined by the SPINO recommendations, independent of its location (bone, epidural space, soft tissues).

**Figure 1:**
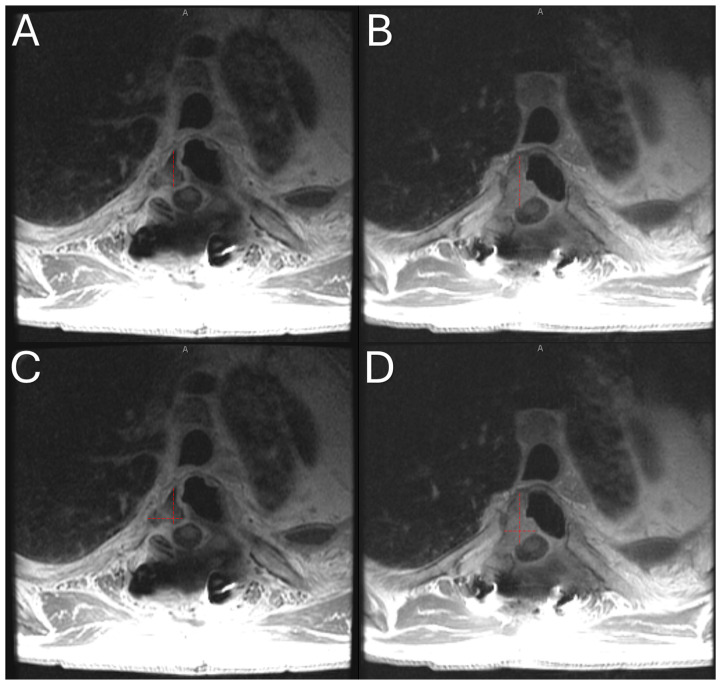
Representative case showing the unidimensional (RECIST1.1 (**A**,**B**)) and bidimensional (MDACC (**C**,**D**)) measurements for a locally progressive metastatic spinal lesion in an axial T1W-MRI at the T4 level, initial (**A**,**C**) and progression (**B**,**D**).

For RECIST1.1 and MDACC criteria, the radiographic-treated lesion was measured by one author for objective definition of response at two timepoints: (1) the baseline MRI obtained after surgery and prior to SSRS, and (2) the MRI demonstrating lesion growth, or the most recent MRI if no growth was seen. Lesions were measured according to each system’s specifications: RECIST1.1 used unidimensional measurement along the longest axis, while MDACC used bidimensional measurement (longest axis plus the longest orthogonal dimension), with response category assignment based on each system’s thresholds for changes in percentage (Table 1, Figure 1).

The above information was adapted from [22,27].

### 2.3. Data Analysis

For the demographic table normality tests for continuous variables, measures of central tendency and dispersion were calculated. Comparisons between groups were made using Chi-square test and analysis of variance (or their non-parametric counterparts as appropriate). Association and agreement analyses, and descriptive survival plots were made using R v4.4.1 [28]. (Event of interest: local progression; time origin: calculated from date of first SSRS session; censoring: lost to follow-up, death, those without local progression.)

To facilitate association and agreement analyses, categories in MDACC and RECIST1.1 were binarized into local progression (progressive disease) and local control (stable disease, partial response and complete response); SPINO response status was used as reference. MDACC and RECIST1.1 were applied when their respective criteria were met; those coded as not evaluable for that framework were excluded from analyses.

Associations were summarized using the phi (φ) coefficient. Calculations were performed for MDACC vs. SPINO-defined local progression and for RECIST1.1 vs. SPINO-defined local progression. Inter-framework agreement between response system (RECIST1.1 vs. SPINO and MDACC vs. SPINO) was quantified using Cohen’s kappa, with unweighted agreement for binary rating. Agreement between RECIST1.1 and MDACC was assessed if overlapping cases were present.

## 3. Results

### 3.1. General Demographic Data

Ninety-four interventions in 93 patients were identified (Table 2). The median age at surgery was 58.9 years (range, 21–81.5 years), 50 (53.2%) were male and 44 (46.8%) females. Metastases were located predominantly in the thoracic (n = 67, 71.3%) and lumbar (n = 15, 16%) spine. Epidural spinal cord compression grades 2 and 3 were present in most cases (n = 76, 80.9%). Sixty-seven patients (71.3%) had radioresistant histologies and 17 (18.1%) patients had previous radiation treatment.

### 3.2. Treatment Data

All patients underwent decompression surgery through a posterior or posterolateral approach and instrumentation with pedicle screws and rods. Thirty-six (38.3%) cases had vertebral body reconstruction with polymethylmethacrylate (PMMA). Postoperative treatment planning using CT myelogram as adjunct was performed in 49 (52.1%) of the cases.

The median time between surgery and SSRS was 5.3 weeks (range, 2.9–12.9) and was delivered in one or three fractions (n = 46 [48.9%] and n = 48 [51.1%], respectively). The median equivalent dose in 2-Gy fractions (EQD2) with an α/β = 10 was 42.8 Gy (range, 36–83.3) and the median biologically effective dose (BED) with an α/β = 10 was 51.3 Gy (range 43.2–99.9).

### 3.3. Outcome Data

Patients were followed for a median time of 16 months (range, 1–132 months). Regarding residual tumor location postsurgery, 85 patients (93.6%) of patients had residual disease after surgery prior to SSRS, and six (6.4%) patients had the lesions completely removed with surgery. Residual disease was confined to bone-only residual disease in 44 (46.8%), bone with soft tissue component (epidural or paravertebral) in 29 (30.8%) cases, isolated residual paravertebral/paraspinal disease was present in twelve (12.8%), and isolated residual epidural disease in three (3.2%).

**Figure 2:**
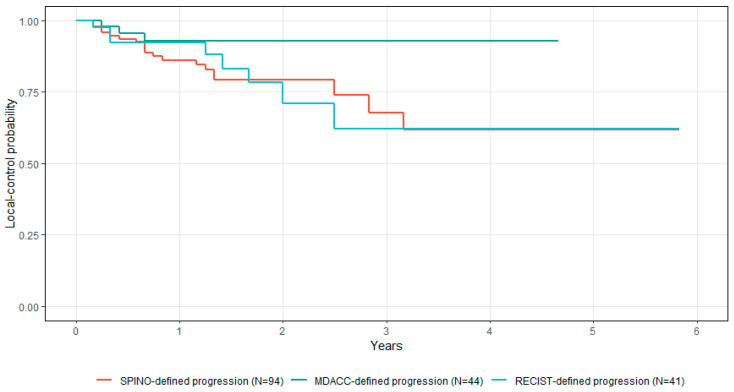
Kaplan–Meier curves show local control probability using local progressive disease definitions from SPINO, MDACC and RECIST1.1. Curves are estimated within each framework’s evaluable subset (SPINO: n = 94, MDACC: n = 44, and RECIST1.1: n = 41). Frameworks do not incorporate the exact patient population and do not classify the same radiographic changes as progression; therefore, statistical analysis was precluded and curves should be interpreted as exploratory only.

The overall local progression rate including any local progression across the three criteria was 21.3% (n = 20) and all progressions were captured with the SPINO recommendations, of which, one was a new lesion identified by PETCT, one did not have change in growth but positron emission tomography identified increased fluorodeoxyglucose (FDG) uptake, and one did not have MRI images available but radiology reported growth of the lesion. Overall local control rate at 1 year for each criterion varied: for SPINO it was 86% (N = 94), for MDACC it was 92.8%, and for RECIST1.1 was 92.1% (Figure 2).

### 3.4. RECIST1.1 Versus MDACC Versus SPINO Performance

All cases had post-SSRS and follow-up MRIs with reports available, except for three, for which the classification relied exclusively on the radiology report. Using SPINO recommendations, response assessment was feasible in 100% (n = 94) of cases, classifying 20 cases as local progression and 74 as local control.

In contrast, RECIST1.1 criteria could be applied to 43.6% (n = 41/94) of this cohort; the remaining 53 patients were not classifiable because 3 had exclusive epidural disease, 6 did not meet criteria for target lesions and 44 had bone metastasis without soft tissue components. MDACC criteria were applied to 46.8% (n = 44/94), with the remaining 50 patients not being able to be classified because 3 were exclusive epidural disease, 18 had no measurable bone metastasis, and 29 had mixed compartment lesion.

Among evaluable cases, RECIST classified 8/41 as progressive disease, 28/41 as stable disease, and 5/41 as partial response. MDACC classified 3/44 as progressive disease and 41/44 as stable disease.

### 3.5. Association Coefficients and Inter-Framework Agreement

MDACC demonstrated strong association (φ = 0.82, n = 44 evaluable cases) when compared with SPINO-based outcomes. In contrast, RECIST1.1 demonstrated negligible association (φ = 0.023, n = 41 evaluable cases). Because RECIST1.1 and MDACC were applicable only to subsets of the cohort, these estimates are subset-specific and may partially reflect differences in evaluability rather than in classification performance alone.

Agreement between SPINO and MDACC classification was perfect (κ = 1, n = 44 evaluable cases); and between SPINO and RECIST 1.1 was poor (κ = 0.07, n = 29 evaluable cases). No cases were jointly evaluable by MDACC and RECIST1.1; therefore, agreement could not be estimated (Table 3).

## 4. Discussion

Radiation is a cornerstone of treatment for spinal metastatic disease. SSRS is an ablative therapy delivered in one to five fractions with higher doses per fraction when compared to conventional radiation. SSRS is typically recommended in oligometastatic disease or progression as an adjuvant to surgery, radioresistant histology, or in cases where previous radiation schemes have failed [8,29].

Stereotactic radiosurgery has consistently demonstrated high rates of local control, which range between 80 and 96%, reported by multiple authors and reviews [2,9,29,30,31,32,33]. A common limitation across studies is the lack of consistent reporting on how radiographic tumor response was assessed. To address this, response assessment tools such as the WHO, RECIST 1.1, and the MDACC criteria—originally developed for nonspinal bone metastases—can theoretically be applied to spinal metastatic disease. However, each of these systems have significant limitations in the ability to classify metastatic spinal tumor given the mixture of bone and soft tissue involvement in spinal metastatic tumors [22,34].

The lack of a widely accepted or validated assessment tool may limit the standardization of response classifications. There have been limited comparisons of different response assessment tools in spine, especially modern tools. Harel et al. compared two of these tools. In 111 diseased vertebral bodies, they measured pre- and post-treatment lesions and categorized them using the WHO and RECIST1.1 criteria. The study concluded that both methods showed significant statistical agreement, although RECIST was favored [35].

In 2015, the SPINO group published the results of an expert survey, providing a set of recommendations to assess tumor response to SSRS in spinal metastatic disease. Their consensus favors MRI as the imaging modality of choice, and the interpretation should be performed by a radiation oncologist and radiologist. The definition of local control is an “absence of progression within the treated area on serial imaging”, and local progression is defined as: 1. gross unequivocal increase in tumor volume or linear dimension, 2. any new or progressive tumor within the epidural space, 3. neurological deterioration attributable to pre-existing epidural disease with equivocal increased dimensions. They also suggest considering pseudoprogression or necrosis mimicking true growth and confirmation with biopsy [27].

Magnetic resonance imaging (MRI)-based follow-up is particularly valuable in patients with metastatic spinal disease treated with surgery and adjuvant SSRS, because it allows detailed assessment of the spinal cord, spinal canal, neural elements, and paraspinal soft tissues [36]. MRI facilitates distinction between local control, true progression, and pseudoprogression, and permits detection/differentiation of postoperative fluid collections, fibrosis, inflammation, and treatment-related bone changes [37]. Current recommendations typically favor MRI every 3 months during the first year after SSRS, with less frequent imaging in subsequent years; this schedule may be further refined by stratifying patients according to their risk of recurrence, as suggested by Chen et al. [27,38]. Despite being the most sensitive and specific modality for local assessment, MRI is not exempt from artifacts related to metallic spinal instrumentation. Its diagnostic yield can be improved by metal artifact reduction techniques such as Slice Encoding for Metal Artifact Correction (SEMAC) and Multi-Acquisition Variable Resonance Image Combination (MAVRIC) [25,26], or by the use of nonmetallic hardware (e.g., carbon fiber-reinforced polyetheretherketone), which has demonstrated improved imaging quality without compromising construct durability or local control [39,40,41].

No currently available spine-focused tool comprehensively incorporates bone, paravertebral/paraspinal, and epidural disease within a unified set of objective response criteria. However, the first SPINO definition of local control—absence of progression within the treated area—offers a flexible conceptual framework for such disease patterns. Two Canadian studies have reported using SPINO recommendations in clinical trials [11,12].

Our work also highlights the potential adjunctive role of metabolic imaging as recommended in the SPINO framework. In two cases from our cohort, PET-based findings contributed to progression classification despite limited size change on MRI. While metabolic imaging is not routinely required, it may be useful when MRI findings are equivocal or when lesion activity is uncertain, complementing anatomic imaging assessment [22].

Overall, our findings support the feasibility and clinical utility of the SPINO framework for response assessment after surgery and adjuvant SSRS. This observation is consistent with our institution’s prior experience applying the SPINO recommendations in separate cohorts to evaluate radiographic surveillance and response characterization in spinal metastasis [42,43,44]. SPINO-based assessment was possible in 100% of our cases, substantially higher than with MDACC (46.8%) and RECIST 1.1 (43.6%), largely because the latter systems do not reliably accommodate soft tissue and epidural disease—patterns that are clinically common and highly consequential in patients treated for ESCC. Consistent with this, association with SPINO-defined progression was strong for MDACC (φ = 0.82; κ = 1) but negligible for RECIST1.1 (φ = 0.023; κ = 0.07), likely reflecting different patient eligibility cohorts based on disease location.

Regarding 1-year local control rates, SPINO yielded a rate of 86.1%, compared with approximately 92% for both MDACC and RECIST 1.1. SPINO provides a global framework applicable to bone, epidural and soft tissue disease. In contrast, MDACC was developed primarily for osseous metastases and emphasizes bone signal/density changes and remodeling, underestimating epidural and soft-tissue components. RECIST1.1 relies on size thresholds for measurable target lesions; requires soft tissue lesions to measure >10 mm, and bone target lesions to be accompanied by a measurable soft tissue component, which may inadequately assess small volume lesions and isolated epidural progression.

Together the differences in applicability and inter-framework agreement highlight the inability of MDACC and RECIST1.1 to incorporate multicompartmental spinal disease, in contrast to the practical and comprehensive framework offered by the SPINO recommendations. The apparent differences in local progression rates across the scoring tools, particularly survival analysis presented in Figure 2, highlight that these differences in applicability may shift the results of commonly reported outcomes such as local progression, rather than reflect treatment efficacy or classification accuracy.

While our findings support the broad applicability of the current SPINO recommendations for evaluating SSRS-treated spinal metastases, it is not without limitations. The binary categorization of SPINO, lack of standardized quantitative measures, and emphasis on radiologist interpretation limits the objectivity and granularity of the data it generates. Future incorporation of standardized measurements, such as those used in RECIST 1.1 and MDACC, into future versions of SPINO may further strengthen its utility.

## 5. Limitations

This is a retrospective, single-institution design which may introduce inherent selection bias, limiting generalizability to broader patient populations. Differences in tools’ applicability preclude adequate statistical analysis for survival; therefore, Kaplan–Meier curves are exploratory only, comparing different evaluable subsets over time rather than an identical patient population. A formal interobserver reliability analysis was not performed; the primary objective of this study was to compare the applicability of three existing response assessment frameworks in a real-world postoperative cohort, rather than validating frameworks against an independent reference standard; therefore, a dedicated interobserver agreement study was outside the scope of this analysis.

## 6. Conclusions

The SPINO recommendations offer a practical and comprehensive guideline to evaluate local control after surgery and SSRS for spinal metastases. This study describes SPINO’s broad applicability in contrast to widely used assessment tools RECIST 1.1 and MDACC.

## Figures and Tables

**Table 1:** Summary and comparison of assessment tools.

	SPINO Group	MDACC	RECIST 1.1
Local Control	Absence of progression within the treated area on serial imaging. *	Complete response: normalization of signal intensity on MRI or bone density on CT, complete sclerotic fill for lytic lesions on CT, or both.	Complete response: disappearance of target lesions.
		Partial response: ≥50% decrease in measurable lesions (subjective for ill-defined lesions), development of sclerotic rim or partial sclerotic fill for lytic lesions on CT, or both.	Partial response: ≥30% decrease in sum of target lesions diameters.
		Stable disease: any response other than complete or partial response and progressive disease.	Stable disease: any response other than complete or partial response and progressive disease.
Local Progression	Gross unequivocal increase in tumor volume or linear dimension. OR Any new or progressive tumor volume within the epidural space. OR Neurological deterioration is attributable to pre-existing epidural disease with equivocal increased epidural disease dimension on MRI.	Progressive disease: ≥25% increase in measurable lesions (subjective for ill-defined lesions).	Progressive disease: ≥20% increase in sum of target lesion diameters + absolute increase of ≥5 mm, appearance of one or more new lesions, unequivocal progression of non-target lesions, or a combination.

* Two or three consecutive MRI scans 6–8 weeks apart.

**Table 2:** Demographic characteristics.

Variable	RECIST (N = 41)	MDACC (N = 44)	SPINO (N = 94)	*p* Value
Applicability in total cohort, %	43.6	46.8%	100%	
Local Progression, n (%)	8	3	20	
Local Control, n (%)			74	
Stable Disease, n (%)	28	41	N/A	
Partial Response, n (%)	5	0	N/A	
Complete Response, n (%)	0	0	N/A	
Sex				0.358
Female, n (%)	16 (39)	24 (54.5)	44 (46.8)	
Male, n (%)	25 (61)	20 (45.5)	50 (53.2)	
Age at surgery, y, median (range)	57 (21–81.5)	59.8 (30–75)	58.9 (21–81.5)	0.83
Fractions				0.07
1, n (%)	14 (34.1)	26 (59.1)	46 (48.9)	
3, n (%)	27 (65.9)	18 (40.9)	48 (51.1)	
EQD2 α/β = 3, Gy, median (range)	64.8 (64.8–129.6)	102.6 (52.8–129.6)	70.2 (52.8–162.0)	0.29
BED α/β = 3, Gy, median (range)	108 (108–216)	171 (88–216)	117 (88–270)	0.29
EQD2 α/β = 10, Gy, median (range)	42.8 (42.8–68)	55.4 (36–68)	42.8 (36.0–83.3)	0.40
BED α/β = 10, Gy, median (range)	51.3 (51.3–81.6)	66.5 (43.2–81.6)	51.3 (43.2–99.9)	0.40
GTV Minimum, cGy, median (range)	1027.1 (668.8–2113.8)	1034.7 (586.2–2736.3)	1025.1 (586.2–2736.3)	0.7
Duration of follow-up, m, median (range)	19 (1–132)	13.5 (3–76)	16 (1–132)	0.32
Length of construct, segments, median (range)	5 (3–10)	5 (2–10)	5 (2–10)	0.49
Time from Surgery to SSRS, w, median (range)	6 (2.9–12.9)	4.6 (3–9.9)	5.3 (2.9–12.9)	0.07
Tumor resistance to radiation				0.96
Radioresistant, n (%)	30 (73.2)	31 (70.5)	67 (71.3)	
No Radioresistant/unclassified, n (%)	11 (26.8)	13 (29.5)	27 (28.7)	
Level of Pathology				0.93
Cervical, n (%)	4 (9.8)	3 (6.8)	7 (7.4)	
Cervicothoracic, n (%)	3 (7.3)	1 (2.2)	4 (4.3)	
Thoracic, n (%)	26 (63.4)	32 (72.7)	67 (71.3)	
Thoracolumbar, n (%)	0	1 (2.2)	1 (1.1)	
Lumbar, n (%)	8 (19.5)	7 (15.9)	15 (16)	
ESCC Scale				0.15
Grade 1c, n (%)	5 (12.2)	13 (29.5)	18 (19.1)	
Grade 2, n (%)	21 (51.2)	24 (54.5)	51 (54.3)	
Grade 3, n (%)	15 (36.6)	7 (16)	25 (26.6)	
Systemic Progression				
Yes, n (%)	38 (92.7)	43 (97.8)	89 (94.7)	
No, n (%)	3 (7.3)	1 (2.2)	5 (5.3)	
CT Myelogram				0.58
Yes, n (%)	18 (43.9)	24 (54.5)	49 (52.1)	
No, n (%)	23 (56.1)	20 (45.5)	45 (47.9)	
Previous Radiation Schemes				0.74
Yes, n (%)	8 (19.5)	6 (13.6)	17 (18.1)	
No, n (%)	33 (80.5)	38 (86.4)	77 (81.9)	
Vertebral Body Reconstruction				0.64
Yes, n (%)	17 (41.5)	14 (31.8)	36 (38.3)	
No, n (%)	24 (58.5)	30 (68.2)	58 (61.7)	

**Table 3:** Evaluability and dichotomized response classification by framework.

Framework	Evaluable, n (%)	Not Evaluable, n (%)	PD, n (%) *	SD, n (%) *
SPINO	94 (100.0%)	0 (0.0%)	20 (21.3%)	74 (78.7%)
MDACC	44 (46.8%)	50 (53.2%)	3 (6.8%)	41 (93.2%)
RECIST 1.1	41 (43.6%)	53 (56.4%)	8 (19.5%)	23 (80.5%)

* Percentages for PD/SD are calculated among evaluable cases for each framework.

## Data Availability

The data presented in this study are available on reasonable request from the corresponding author due to privacy restrictions.

## Abbreviations

The following abbreviations are used in this manuscript:

BED	Biologically Effective Dose
CT	Computed Tomography
CTV	Clinical Target Volume
EBRT	External Beam Radiotherapy
EQD2	Equivalent Dose in 2-Gy Fractions
ESCC	Epidural Spinal Cord Compression
FDG	Fluorodeoxyglucose
GTV	Gross Tumor Volume
MAVRIC	Multi-Acquisition Variable Resonance Image Combination
MDACC	MD Anderson Cancer Center
MRI	Magnetic Resonance Imaging
NOMS	Neurologic–Oncologic–Mechanical–Systemic Framework
PET	Positron Emission Tomography
PMMA	Polymethylmethacrylate
RECIST	Response Evaluation Criteria in Solid Tumors
SEMAC	Slice Encoding for Metal Artifact Correction
SSRS	Stereotactic Spine Radiosurgery
SINS	Spinal Instability Neoplastic Score
SPINO	Spine Response Assessment in Neuro-Oncology Group
UICC	International Union Against Cancer
VB	Vertebral Body
WHO	World Health Organization
